# Mechanisms Relevant to the Enhanced Virulence of a Dihydroxynaphthalene-Melanin Metabolically Engineered Entomopathogen

**DOI:** 10.1371/journal.pone.0090473

**Published:** 2014-03-24

**Authors:** Min-Nan Tseng, Chia-Ling Chung, Shean-Shong Tzean

**Affiliations:** 1 Department of Plant Pathology and Microbiology, National Taiwan University, Taipei, Taiwan; 2 Division of Plant Protection, Kaohsiung District Agricultural Research and Extension Station, Council of Agriculture, Executive Yuan, Pingtung, Taiwan; Natural Resources Canada, Canada

## Abstract

The entomopathogenic fungus *Metarhizium anisopliae* MA05-169 is a transformant strain that has been metabolically engineered to express dihydroxynaphthalene-melanin biosynthesis genes. In contrast to the wild type strain, the transformant displays a greater resistance to environmental stress and a higher virulence toward target insect host. However, the underlying mechanisms for these characteristics remain unclear; hence experiments were initiated to explore the possible mechanism(s) through physiological and molecular approaches. Although both transformant and wild type strains could infect and share the same insect host range, the former germinated faster and produced more appressoria than the latter, both *in vivo* and *in vitro*. The transformant showed a significantly shorter median lethal time (LT_50_) when infecting the diamondback moth (*Plutella xylostella*) and the striped flea beetle (*Phyllotreta striolata*), than the wild type. Additionally, the transformant was more tolerant to reactive oxygen species (ROS), produced 40-fold more orthosporin and notably overexpressed the transcripts of the pathogenicity-relevant hydrolytic enzymes (chitinase, protease, and phospholipase) genes i*n vivo*. In contrast, appressorium turgor pressure and destruxin A content were slightly decreased compared to the wild type. The transformant's high anti-stress tolerance, its high virulence against five important insect pests (cowpea aphid *Aphis craccivora*, diamondback moth *Pl. xylostella*, striped flea beetle *Ph. striolata*, and silverleaf whitefly *Bemisia argentifolii*) and its capacity to colonize the root system are key properties for its potential bio-control field application.

## Introduction

While the entomopathogenic fungi, including *Beauveria*, *Metarhizium* et al., have been widely studied and utilized as control agents for pests for decades, their low tolerance to ultraviolet (UV) damage and low control efficacy have remained significant concerns for field applications [1]–[4]. A lack of melanin to circumvent environmental stresses may partially explain their low biocontrol efficacy in the field [1], [3]–[5]. Thus, in a previous study aiming at enhancing the applicability of *Metarhizium* spp., we introduced genes of the melanin biosynthesis pathway from *Alternaria alternata* into the wild-type *M. anisopliae* strain BCRC35505. The transformant strain *M. anisopliae* MA05-169, which is capable of expressing exogenous dihydroxynaphthalene melanin (DHN-melanin), exhibited a greater tolerance to UV-irradiation, high temperatures, and desiccation and a higher virulence toward *Plutella xylostella* [5]. Because melanin is well known for its role in protecting organisms against environmental stresses [6], [7], the increased stress tolerance observed in *M. anisopliae* 05-169 is expected. The enhanced virulence of the melanized transformant MA05-169 on *P. xylostella*, in contrast, brought up some interesting questions - Is melanin associated with the enhanced virulence? If so, at which stage(s) of pathogenesis is melanin playing a role? Finally, what are the physiological, biochemical and/or molecular mechanisms involved in the superior performance of this melanin-producing strain?

Several studies have revealed the pathogenesis of entomopathogenic fungi and the interactions between entomopathogenic fungi and their insect hosts during the process of infection. Infection with an entomopathogenic fungus is initiated when the conidium adheres to the insect cuticle by means of hydrophobicity and static electricity [8]. Hydrophobins, a class of structural proteins of the fungal spore surface, are known to be involved in the nonspecific attachment of the conidium to the hydrophobic surface of the insect cuticle [9]. Some enzymes are also involved in conidia adhesion to the insect cuticle. The activities of ecto-phosphatase and glyceraldehyde-3-phosphate dehydrogenase (GAPDH) also affect the adhesive capability of spores [9]–[11]. MAD1, an adhesin-like protein generated after spore germination, mediates stronger and more specific cohesion between *Metarhizium* spp. and their insect hosts [12]. In entomopathogenic fungi, the germination speed and adhesive capability of spores have been found to be positively correlated with virulence [13], [14]. Using the conidia of *M. anisopliae* from different nutrient sources, Shah et al. (2005) observed a positive correlation between the germination speeds and the virulence of *Galleria mellonella* and *Tenebrio molitor* [15]. Altre and Vandenberg (2001) also found that for *Paecilomyces fumosoroseus*, the level of virulence was correlated with the conidial germination speed on the external surface of *Lepidopteran* [16].

Following adhesion, the conidium germinates and forms an appressorium for penetration, although infection can also be achieved by direct penetration of the extended hyphal tip. The appressorium produces adhesive substances, enabling it to adhere firmly to the host body [17], [18]. The appressorium secretes hydrolytic and lipolytic enzymes, such as proteases, chitinases, lipases, and esterases, to decompose the epidermal structure of the host [19]. With the aid of the enormous turgor pressure generated inside the appressorium due to water entering the cell by osmosis, the emerging infection peg uses mechanical force to penetrate the weakened insect cuticle.

Entry into the host is just the beginning of the infection process. After penetration, the invasive hyphae of the entomopathogenic fungus first encounter the immune system of the insect. In general, pathogenic fungi that invade animal bodies are subject to attacks by large numbers of immune cells, such as monocytes and macrophages in humans and hemocytes in invertebrates. Only the pathogenic fungi that successfully escape or resist immune attacks have the opportunity to colonize the host body and cause diseases [7], [20]. Nodulation, phagocytosis, and encapsulation are immune responses that are commonly observed in animal systems. For instance, in the human body, the foreign microbe detected by the immune cell may be engulfed via pinocytosis and then put into the phagocytic vacuole. In the phagocytic vacuole, the activated nicotinamide adenine dinucleotide phosphate hydrogen oxidase (NADPH oxidase) catalyzes the reduction of molecular oxygen into peroxide and superoxide (O_2_^−^). The O_2_^−^ subsequently reacts with nitric oxide (NO) to produce peroxynitrite (ONOO^−^) [7]. Myeloperoxidase (MPO) also enters the phagocytic vacuole, using the Cl in the vacuole to generate HOCl. Among the diverse defensive responses in an animal cell, the rapid generation of ^ ˙^O_2_^−^ and H_2_O_2_ by NADPH oxidase is crucial for establishing early resistance against the pathogen. The virulence of a pathogenic fungus is thus considered to be associated with its ability to directly or indirectly affect the production of O_2_^−^ or H_2_O_2_ in the host cell [21].

The innate immune system of insects resembles that of mammals [22]. Among the various types of hemocytes in the insect haemocoel, plasmatocytes and granulocytes have features similar to macrophages in vertebrates. Upon contact with non-self antigens such as bacteria or fungi, hemocytes engulf foreign objects and trigger oxidative metabolism, which transforms oxygen into antimicrobial reactive oxygen intermediates (ROIs). For example, in *Drosophila melanogaster*, the generation of O_2_^−^ was observed during the melanotic encapsulation of the parasitic *Leptopilina boulardi*. In superoxide dismutase (SOD)- and catalase (CAT)-deficient *D. melanogaster*, the immunodeficiency disorders were partially attributed to a defect in H_2_O_2_ metabolism [23]. In *Anopheles gambiae*, nitric oxide proved to be a key component of the hemocyte-mediated immune system [24].

Melanin is known to contribute to the stress tolerance and virulence of many fungi - examples include the human pathogenic fungus *Cryptococcus* [25] and the plant pathogenic fungi *Colletotrichum* sp., *Alternaria* sp., and *Magnaporthe* sp. [26]. In plant pathogenic fungi, the main contribution of melanin to virulence is the formation of a melanin layer on the appressorium between the inner cell wall and the plasma membrane, which permits only water and small molecules to enter. Triacylglycerol and glycogen accumulated in the appressorium enable extracellular water to enter the appressorium, forming an enormous turgor pressure that serves as the necessary mechanical force for invasion [25], [27]. To survive and multiply in the animal body, a successful pathogenic microbe must develop ways to minimize the free radical damage caused by the animal immune system. It has been reported that in some species of pathogenic animal fungi, melanin acts as a free radical scavenger [28]. For instance, melanin production in *C. neoformans*, *Aspergillus* spp., *Sporothrix schenckii*, and *Fonsecaea pedrosoi* has been associated with increased tolerance to H_2_O_2_ and to the damage caused by nitrogen or oxygen free radicals [29]–[31]. In addition to the free radical–eliminating property, melanin also protects fungal pathogens against antimicrobial peptides [32], allowing the pathogens to overcome the host immune responses.

As a follow-up to our previous work on the development of a melanin-producing *M. anisopliae*, this study aims to uncover the potential cause(s) of the enhanced virulence of the melanized transformant, *M. anisopliae* MA05-169, on the insect host *P. xylostella*. Because virulence is a complex trait that is determined by numerous factors, we used a series of histological and molecular approaches to examine different aspects of the interactions between entomopathogenic fungi and their insect hosts. To analyze the effect of melanin on the degree of virulence we examined the melanized *M. anisopliae* transformant MA05-169 and the wild-type BCRC35505 under *in vitro* and/or *in vivo* conditions. We compared conidial germination, appressorium formation, and appressorial turgor pressure generation and their ability to resist free radical damage. To better understand the underlying mechanisms, changes in gene expression and secondary metabolites production in *M. anisopliae* MA05-169 and BCRC35505 were further investigated by real-time RT-PCR, liquid chromatography–mass spectrometry (LC-MS), and nuclear magnetic resonance (NMR) analysis.

## Materials and Methods

### Inoculum preparation

The mycelial disc of either the melanized transformant MA05-169 or the wild-type BCRC35505 of *M. anisopliae* was inoculated onto a potato dextrose agar (PDA) plate and incubated in darkness at 28°C for 21 days. The spores harvested by vortexing the agar blocks in sterile 0.1% Tween 80 for 2 min were filtered through Miracloth (EDM Millipore) and counted under a light microscope with a hemocytometer. Unless otherwise indicated, the concentration of the spores was adjusted to 10^7^ conidia ml^−1^ for the inoculation.

### Insect host range assay

The following eight insect species were subjected to tests of their susceptibility toward the melanized transformant (MA05-169) and wild-type (BCRC35505) *M. anisopliae*: cabbage worm (*Pieris rapae*), cowpea aphid (*Aphis craccivora*), diamondback moth (*Plutella xylostella*), oriental fruit fly (*Bactrocera dorsalis*), rhinoceros beetle (*Allomyrina dichotoma*), silverleaf whitefly (*Bemisia argentifolii*), striped flea beetle (*Phyllotreta striolata*), and tobacco cutworm (*Spodoptera litura*). Conidial suspensions (10^7^ conidia ml^−1^) of *M. anisopliae* transformant or wild type were used as inoculums. Sterilized 0.1% Tween 80 was used as control. Unless otherwise indicated, the inoculated insects were incubated at 25°C with a 12∶12 h light∶dark cycle; and each bioassay was repeated three times.

The inoculation procedure for *Pl. xylostella* followed a previously described method [5]. Briefly, groups of twenty synchronized third-instar *Pl. xylostella* feeding larvae were immersed in 5 ml of spore suspension for 30 s. The excess suspension was removed by filter paper. The inoculated larvae were reared on fresh canola leaflets and incubated for 3 days. Groups of ten 2nd-instar *S. litura* or *Pi. rapae* larvae were inoculated by spraying with 1 ml of conidial suspension. After inoculation, all larvae were raised on fresh canola leaflets in a plastic box (12×12×10 cm). For *Al. dithotomus*, the abdomens of 2nd-instar larvae were inoculated by applying 500 µl of conidial suspension. Thereafter, each larva was separately reared on compost in a plastic box (12×12×10 cm) at 25°C in the dark. Each experiment consisted of ten replicate larvae. For *Ap. craccivora*, the stock culture was maintained on asparagus bean (*Vigna unguiculata*) seedlings. Ten *Ap. craccivora* adults were transferred from the stock culture with a painting brush onto a fresh asparagus bean leaf and reared for one more day. Subsequently, except for 20 of the nymphs per leaf, all aphids were removed with a pair of fine tweezers. The 2nd-instar nymphs were inoculated by spraying with 1 ml of conidial suspension. For *Ba. Dorsalis*, groups of ten adults that completed eclosion on the same day were first anesthetized with CO_2_ and then inoculated by spraying with 2 ml of conidial suspension. Thereafter, the insects were confined to a plastic box and reared on a nutrient solution containing peptone, yeast extract, and sucrose in a ratio of 1∶1∶4. For *Ph. striolata*, groups of twenty adults, were inoculated by allowing the insects to crawl for 5 minutes over 3-week-old culture plates containing either the transformant or the wild type *M. anisopliae* on. The inoculated striped flea beetles were then reared on 2–3 rape (*Brassica campestris*) leaves in a Petri dish. For *Be. Argentifolii*, nymphs were reared on the leaves of strawberry (*Fragaria ananassa*) seedlings, followed by spraying with 1 ml of conidial suspension. Observations were performed daily to record the numbers of insects that succumbed, whose body are characterized by turning red, collapsed or covered with mycelium, in contrast, the healthy insects are full and slightly transparent to pale yellow, thus can be distinguished. The infected nymphs were incubated in a moistened Petri dish to examine the development and sporulation of the pathogens on their cadavers.

### Assays of conidial germination and appressorium formation *in vitro*

Twenty microlitres of *M. anisopliae* MA05-169 or BCRC35505 conidial suspensions (10^4^ conidia ml^−1^) were dropped onto the surfaces of 0.188 mm mylar polyester film (DuPont Teijin Films™) and then incubated in a Petri dish moistened with sterile water for 16, 20, 24, 48, and 64 h at 25°C in the dark. The mylar polyester films were transferred to a slide, mounted with a coverslip, and examined under light microscopy at 400× magnification to determine the rate of conidial germination and appressorium formation. A conidium with a germ tube that exceeded its length was counted as germinated. The tip of the germ tube that was distinctly swollen was labeled as the appressorium. Each experiment involved three replicates; in each replicate, at least 100 spores were counted. The germination rate and the appressorium formation rate were calculated by the following equations:
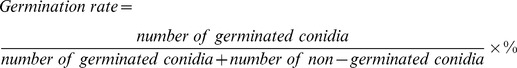


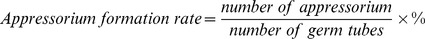



### Assays of conidial germination and appressorium formation *in vivo*

To investigate the conidial germination and appressorium differentiation of *M. anisopliae* transformant and wild type *in vivo*, the inoculation methods and incubation regimes were mainly the same as described above, unless otherwise indicated. Newly molted 3rd-instar *Pl. xylostella* larvae were immersed in spore suspensions of *M. anisopliae* MA05-169 or BCRC35505 for 30 sec. The inoculated larvae were raised on fresh cabbage (*Brassica oleracea*) leaflets and incubated for 20 h at 27°C. *Ap. craccivora* adults and oriental fruit flies (*Ba. dorsalis*) were sprayed with a conidial suspension, whereas *Ph. striolata* adults were inoculated by allowing the adults to crawl over the sporulated colony of 3-week-old culture plates of either the transformant or wild type for 5 minutes on. After inoculation of the cuticle, the abdomen tergites and abdomen sternites of the insects were then removed. The dissected cuticle was mounted on a slide with one drop of 0.1% Calcofluor white, which was added before applying a coverslip. The slide was allowed to sit for 2 min and was then examined under fluorescence microscopy (Leica DFC 480) at 1000× magnification by a built-in excitation filter at 405 nm and an emission filter at 465 nm. A conidium with a germ tube that exceeded its length was counted as germinated. The tip of the germ tube that distinctly swelled was labeled as the appressorium. Each experiment involved three replicates; in each replicate, at least 100 spores were counted for germination rate evaluation, and at least 50 germinated spores were evaluated for appressorium differentiation. The germination rate and the appressorium formation rate were calculated using the equations described above.

### Virulence assay

To assay the virulence of both the transformant and wild-type strains, *Ap. craccivora*, *Ba. dorsalis*, and *Ph. striolata* hosts were inoculated with a conidial suspension of 10^7^ conidia ml^−1^ of the *M. anisopliae* transformant or the wild type. The insects were also sprayed with sterile 0.1% Tween 80 solution as a control. The inoculated insects were then incubated under the conditions described above.

Approximately 15 *Ap. craccivora* adults were reared on an asparagus bean leave that had previously been rinsed with dH_2_O and drained. After 24 h, all adults were removed, leaving behind 20 nymphs per leaf. The 2nd-instar nymphs then were sprayed with 1 ml of conidial suspension, and insects that succumbed were recorded once every 12 h. The same protocol was applied to *Ph. striolata* collected from bok choy (*Brassica rapa* var. *chinensis*) in the field. A group of twenty insects was used, and they were inoculated with 1 ml of conidial suspension. The inoculated insects were reared on bok choy leaves and evaluated for susceptibility as above. For *Ba. Dorsalis*, eclosed adults, in groups of twenty, were anesthetized with CO_2_ and then inoculated with 2 ml of conidial suspension. Following inoculation, the insects were reared under the same conditions and evaluated for susceptibility as described above. This experiment was conducted in three independent trials, each containing three replicates with 20 insects per treatment. The LT_50_ (median lethal time) values were calculated using the Probit regression model to fit the time-mortality data of larvae over 24–72 hrs. The Probit regression was performed using Predictive Analysis Suite Workstation (PASW) statistical software (Version 18.0, Chicago: SPSS Inc.).

### Appressorial turgor pressure assay

To evaluate the turgor pressure of the appressorium of the *M. anisopliae* melanized transformant and the non-melanized wild type, the spore suspension was dropped onto a cricket's (*Gryllus bimaculatus*) hind wings, which were then incubated in moist chambers for 24 h at 25°C in the dark. Individual wings were immersed in a serial concentration of polyethylene glycol 8000 solutions (2, 4, 6, 8, 10, and 12 g in 10 ml of distilled water) for 10 min, and the percentage of collapsed appressoria was determined under light microscopy at 400× magnification [33]. Each experiment involved three replicates; in each replicate, at least 100 appressoria were counted. The collapsed rate was calculated by the following equation:
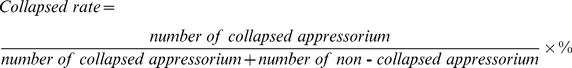



### Assay of damage to *M. anisopliae* by exogenous H_2_O_2_ or nitric oxide

To determine the deleterious effect of hydrogen peroxide (H_2_O_2_) on *M. anisopliae*, conidia were first harvested by washing 3-week-old culture plates of transformant or wild type strains with 100 mM PBS (pH 7.0, containing 0.05% Tween 80).The conidial suspension was adjusted to 10^7^ conidia ml^−1^ in 0, 20, 40, or 60 mM H_2_O_2_ and then incubated at 25°C for 20, 40, or 60 min. Subsequently, the conidial suspension was diluted with 10 mM PBS and spread onto PDA plates. After incubation for 20 h at 25°C, the conidia germination rate was estimated and calculated as described above. In contrast, to determine the detrimental effects of nitric oxide, 2 ml of a conidial suspension (10^5^ ml^−1^) in potato dextrose broth was added to 20 µl of 100, 300, 500, or 600 mM sodium nitroprusside and incubated at 25°C in the dark for 16 h with shaking (150 rpm). Following centrifugation at 10,000 g for 3 min to remove the supernatant, the conidia were resuspended in 100 µl of sterile dH_2_O. The retrieved 10 µl of conidial suspension was mounted on a glass slide, and the germination rate was determined under light microscopy as described above. Each experiment involved three replicates and at least 100 spores were counted in each replicate.

### Effect of melanin on the virulence of transformant and wild type *M. anisopliae*

A 3-ml spore suspension (10^7^ conidia ml^−1^) of *M. anisopliae* transformant MA05-169 or wild type BCRC35505 was centrifuged for 3 min at 10,000 g. The supernatant was removed, and the conidia were resuspended in 3 ml of a solution containing 100 ppm tricyclazole (a melanin biosynthesis reductase inhibitor).

To investigate the virulence of *M. anisopliae* MA05-169 and BCRC35505 for *Pl. xylostella*, synchronized third-instar feeding larvae were inoculated by dipping them into spore suspensions of *M. anisopliae* MA05-1169 or BCRC35505 with or without 100 ppm tricyclazole for 30 s (four treatments in total). The excess suspension was removed with filter paper. The inoculated larvae were reared on fresh canola leaflets and incubated at 25°C for 3 days. The larvae treated with 100 ppm tricyclazole solution without spores were used as the negative control. The experiment was conducted in three independent trials, each containing three replicates with 30 larvae per treatment. The LT_50_ (median lethal time) values were calculated using the Probit regression model to fit the time-mortality data of the larvae over 24–72 hrs.

### Analysis of destruxin and relevant secondary metabolites

To compare the destruxin and relevant secondary metabolites produced by the transformant or wild type, 3 ml of conidial suspension (10^7^ conidia ml^−1^) was inoculated into 200 ml of Sabouraud dextrose broth (SDB) (Difco) in a 1.5 L flask and incubated at 25°C for 10 days with agitation (270 rpm). The mycelium was removed with tissue paper (Kimwipes), and the filtrate was mixed with an equal volume of ethyl acetate. Following rigorous agitation for 2 min, the contents were sonicated in a water bath sonicator for 15 min, and the sonication procedure was repeated six times. Subsequently, the supernatant was condensed with a vacuum rotary evaporator (Eyela Aspirator A-3S, Tokyo, Japan). The dried residue was dissolved in 5 ml of methanol, filtered through a 0.45 µm Millipore membrane, and stored in a brown bottle at −20°C until analysis.

One milligram of authentic destruxin A or cytochalasin C and D (Sigma) was dissolved in 1 ml of methanol to prepare a stock solution, which was serially diluted to 0.125, 0.25, 0.5, and 1 mg/ml to plot the standard curve with Shimadzu HPLC Class-VP™ software v.5.03. Destruxin A was analyzed on a Shimadzu LC-20 series HPLC system equipped with a C18 reverse phase column (SunFire, length: 4.6×150 mm) and detected using UV 215 nm or a photo diode array detector. The linear gradient of the mobile phase of HPLC consisted of H_2_O and acetonitrile. The chromatographic conditions were set as follows: H_2_O:acetonitrile (100∶0) for 0–2 min; 96∶4 to 94∶6 for 6–12 min; 94∶6 to 88∶12 for 12–25 min; 88∶12 to 40∶60 for 25–35 min; 40∶60 for 35–37 min; 40∶60 to 100∶0 for 37–38 min; and 100% H_2_O for 7 min of elution. Twenty microliters of the solvent extract was diluted 100 times with methanol and injected into the column. The eluted destruxin A was monitored at UV 215 nm at a flow rate of 1 ml/min, and the purified compound was further analyzed by liquid chromatography–mass spectrometry (LC/MS) and nuclear magnetic resonance (NMR) [34]. The NMR spectroscopic analysis was performed with a BioSpin AVANCE 400 MHz spectrometer (^1^H 400 MHz, ^13^C 100 MHz, Bruker). In the ^1^H and ^13^C NMR, CD_3_OD was used as the solvent.

### Differential expression of the cuticle hydrolytic enzymes genes *in vivo*

Extracellular chitinase Chit1 (DQ011865.1), subtilisin-like protease Pr1D (AJ400706.1), and phospholipase C (GL698736) were thought to be correlated with the virulence toward insect hosts [33], [35], [36]. Therefore, the sequences of these genes were accessed from the NCBI GenBank database and used for the design of TaqMan probes and oligonucleotide primers (Table 1) for absolute quantitative PCR (AQ-PCR). The AQ-PCR standard curves and the copy number of the gene transcripts of the transformant and wild type on the *Pl. xylostella* insect host were plotted and calculated according to a previously described method [5].

**Table 1:** Primers and probes for real-time polymerase chain reaction (PCR) and reaction conditions.

Genes	Primers & Probe	Annealing temperatures*
Chitinase *chit1*	Chitinase-qPCR-sense: 5′ GCTCAGTCACTGGCAATCATC 3′	57°C
	Chitinase-qPCR-anti2: 5′ ACGTAACCACCGGCA TGTTT 3′	
	Chitinase MGB probe:5′ CCACTCTCGGTCTTGCCACTCCTGT 3′	
Subtilisin-like protease *pr1D*	Protease Pr1D-qPCR-sense: 5′ GGCGTTCTGAGCGTTATTGC 3′	54°C
	Protease Pr1D-qPCR-anti: 5′ CCATTGGGCCAGTTCGTGAC 3′	
	Protease Pr1D MGB probe: 5′ TCTCCAGTTCTTGTCCACGGCACC 3′	
Extracellular phospholipase C	Phospholipase C-qPCR-sense: 5′ AGGCGGCAATCCATATATTGATAAC 3′	57°C
	Phospholipase C-qPCR-anti: 5′ CCTGAAACACATTCCAAGACACC 3′	
	Phospholipase C MGB probe: 5′ TCGGCAACAGTCTTCCACTTGAGCG 3′	

*PCR conditions: denature at 94°C for 3 min, 1 cycle; denature at 94°C for 1 min, anneal at 57°C for 1 min, and extension at 72°C for 2 min, 30 cycles; elongation at 72°C for 5 min, 1 cycle.

Groups of thirty newly molted 3rd-instar larvae of the *Pl. xylostella* were immersed in a spore suspension (10^8^ conidia ml^−1^) of *M. anisopliae* MA05-169 or BCRC35505 for 30 s; the larvae were also immersed in sterile 0.1% Tween 80 as a control. Excess moisture on the inoculated larvae was absorbed with filter paper, and the larvae were reared on fresh cabbage leaflets and incubated at 27°C for 16, 24, 48, and 96 h. The larvae were then ground to a fine power in liquid nitrogen with a mortar and pestle. Total RNA was isolated using an RNeasy mini kit (Qiagen) and reverse transcribed to cDNA using an iScript cDNA synthesis kit (Bio-Rad). The cDNA samples were then subjected to AQ-PCR analyses using the primers, probes, and PCR conditions described above (Table 1).

### Colonization and survival of transformant and wild type *M. anisopliae* on the rhizoplane

Initially, to verify the absence of *M. anisopliae* in the loam soil of the orchard garden collected from Pingtung, Taiwan, that was used for the cultivation of cabbage seedlings, the DNA was extracted from 0.25 g of soil by an UltraClean Soil DNA Isolation Kit (Mo. Bio. USA), according to the manufacturer's instruction. The DNA extracted from the soil samples determined ranged from 16 to 29 ng µl ^−1^µg^−1^ soil_._ The DNA concentration was diluted to 10 ng µl ^−1^ and use 2 µl as a template for PCR. Whereas DNA extracted from the 0.25 g of loam soil samples containing 1 ml of a spore suspension (1×10^6^) of either *M. anisopliae* BCRC35505 or MA05-169 were used as a positive check. The PCR universal primers (8F 5′-GTTTGATCCTGGCTCAG-3′, 1512R 5′-GGYTACCTTGTTACGACTT3′), (ITS4 5′-TCCTCCGCTTATTGATATGC-3′, ITS5 5′-GGAAGTAAAAGTCGTAACAAGG-3′) or specific primers (PKS-TE(a), PKS-TE(b)) were adopted from previously described methods [5]; the two-step nested PCR procedure described by Entz et al. (2008) [37] was followed to detect the *M. anisopliae* in the soil. In brief, in the 1st step of the nested PCR procedure, the DNA segment between the large subunit rDNA and the intergenic spacer (IGS) region was amplified using primers Ma-28S4 (5′-CCTTGTTGTTACGATCTGCTGAGGG-3′) and Ma-IGS1 (5′-CGTCACTTGTATTGGCAC-3′). The reaction mixture contained 10 µl of DNA polymerase (GoTaq Green Master Mix, Promega, USA), 1 µl each of the Ma28S4 and Ma-IGS1 primers, 2 µl of DNA template (20 ng), and 6 µl of ddH_2_O. The thermocycle conditions for the 1st step of the nested PCR were set as follows: Initial denaturation at 94°C for 5 min, 1 cycle; denaturation at 94°C for 1 min, annealing at 54°C for 1 min, and extension at 72°C for 2 min, 30 cycles; and final extension for 5 min, 1 cycle. One µl of the first PCR product was used as the 2nd-step nested PCR template using Ma-IGSspF (5′-CTACCYGGGAGCCCAGGCAAG-3′) and Ma-IGSspR (5′-AAGCAGCCTACCCTAAAGC-3′) primers. The conditions for the 2nd-step nested PCR were the same as those of the 1st-step nested PCR, except that the annealing temperature was instead set to 58°C.

For the detection of the colonization and survival of *M. anisopliae* transformant (MA05-169) and wild type (BCRC35505) on the rhizoplane, seedlings planted on micro-plot loam soil at the two-leaf stage (approximately 4 weeks old) were inoculated with 1 ml of a conidial suspension (1×10^6^ conidia ml^−1^) of both strains, in contrast, inoculated with a sterile 0.1% Tween 80 as a negative control. The inoculated seedlings were incubated in a greenhouse under the natural light-dark cycle and at an ambient air temperature of 28–34°C. After inoculation, the seedlings were excavated every week for 5 successive weeks, and their roots were thoroughly rinsed three times with sterile water to remove soil debris. A root segment of approximately 4 cm in length was plated out on semi-selective Sabouraud dextrose agar containing 10 mg ml^−1^ dodine, 50 mg ml^−1^ chloramphenicol, and 200 mg ml^−1^ cycloheximide [38], and incubated for 20 days at 25°C in the dark. The cultures derived from a single colony were subcultured to PDA. The DNA extracted from the one-week-old culture [5] was subsequently used as a template for a two-step nested PCR, using the Ma-26S4 and Ma-IGS1 primers in the 1st step and the Ma-IGSspF and Ma-IGSspR primers in the 2nd step. In addition, the PKS-TE (a) and PKS-TE (b) specific primers were used to confirm the identity of the *M. anisopliae* transformant MA05-169 culture. The direct PCR protocol was the same as previously described [5].

### Statistics

Unless otherwise indicated, all data were analyzed with a one-sample *t*-test at *P*<0.05 using the Predictive Analysis Suite Workstation (PASW) statistical software (Version 18.0, Chicago: SPSS Inc.).

## Results

### Insect host range

**Figure 1:**
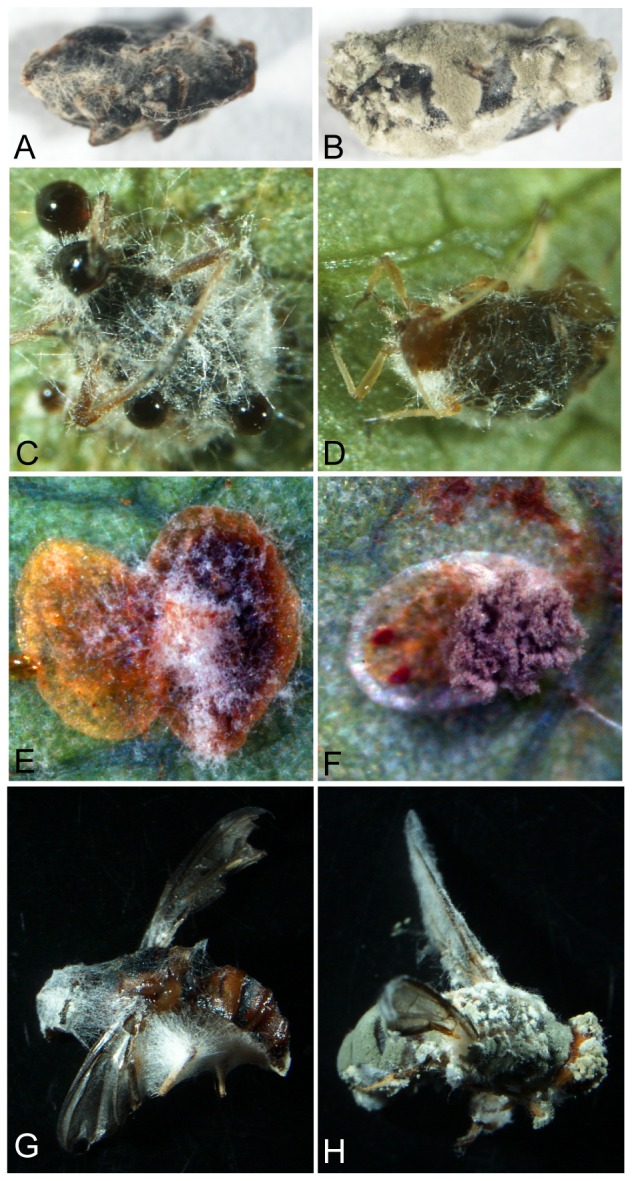
Inoculation and infection of striped flea beetle (*Phyllotreta striolata*) (A–B), aphid (*Aphis craccivora*) (C–D), silverleaf whitefly (*Bemisia argentifolii*) (E–F), and oriental fruit fly (*Bactrocera dorsalis*) (G–H) by *Metarhizium anisopliae* transformant (MA05-169) (A, C, E, G) and wild type (BCRC35505) (B, D, F, H). Abundant sporulation, deep-pigmented droplet exudates, and mycelium covering the carcass are visible.

**Figure 2:**
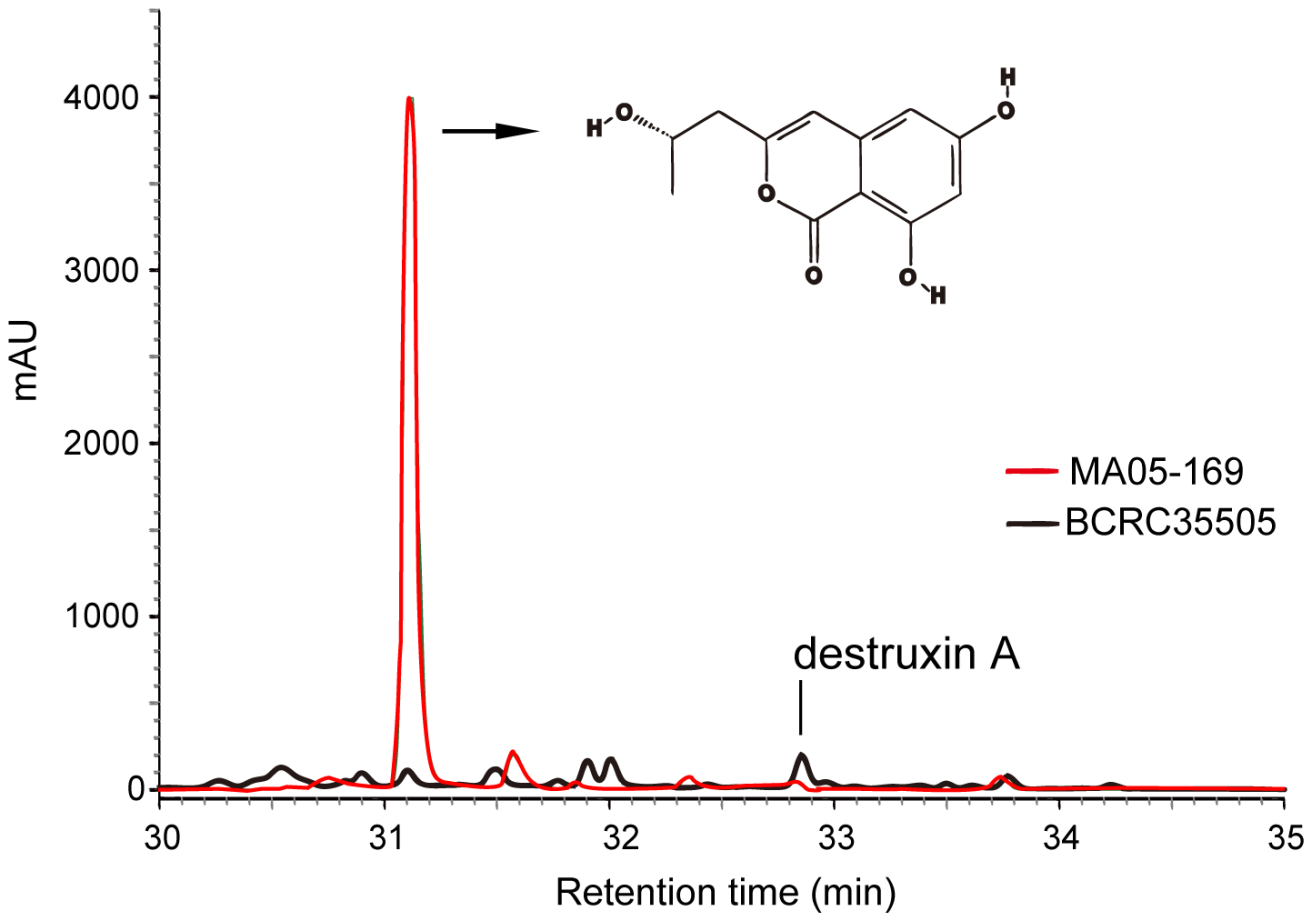
The distinct red peak indicates the de-O-methyldiaporthin (orthosporin) extracted from *Metarhizium anisopliae* transformant (MA05-169), in contrast to the indistinct peak obtained from the wild type strain (BCRC35505). The arrow indicates the molecular structure of orthosporin. Instead, the wild type (BCRC35505) produce 3.2 folds more destruxin A than the *M. anisopliae* transformant (MA05-169).

**Figure 3:**
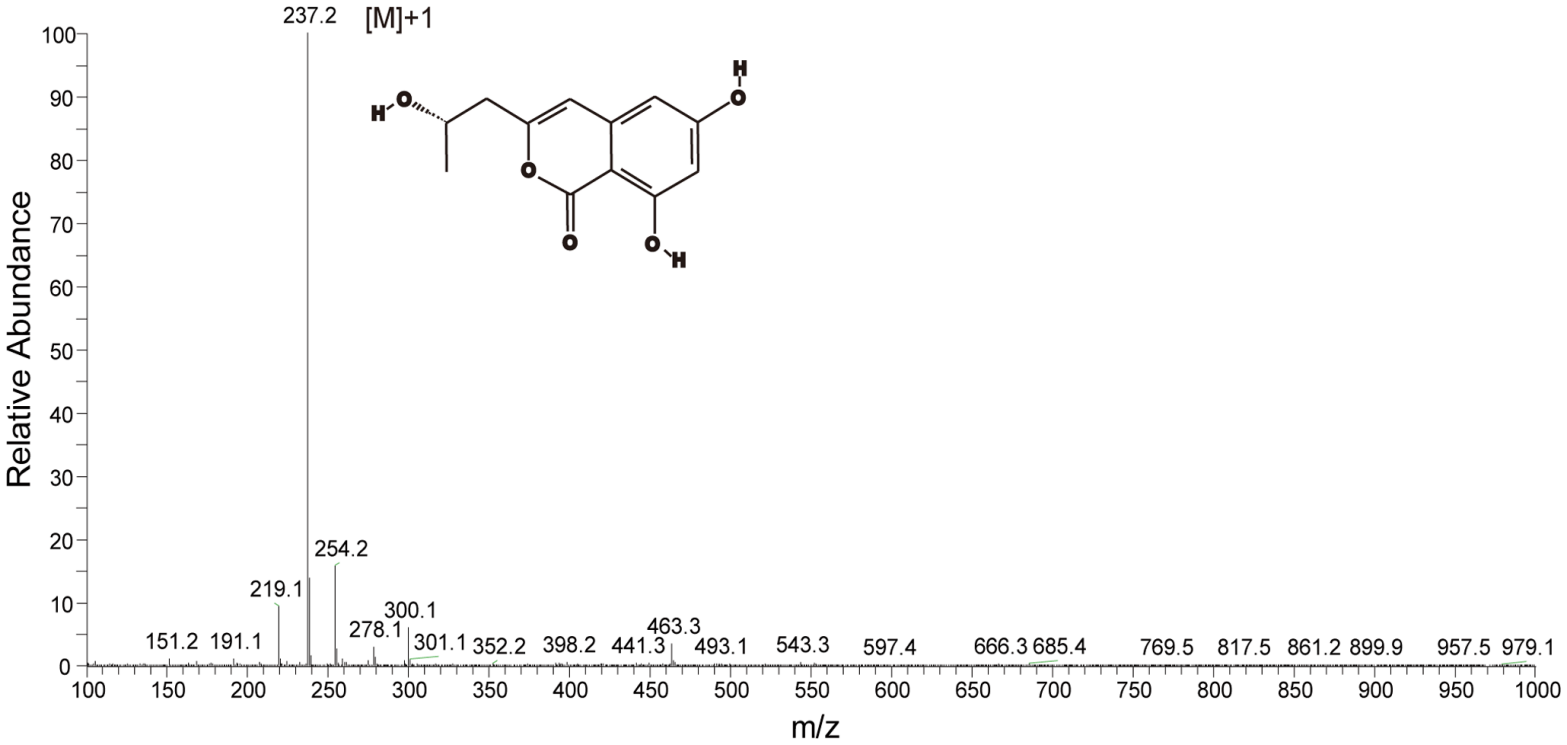
The LC/MS graph of de-O-methyldiaporthin (orthosporin) extracted from the *Metarhizium anisopliae* transformant (MA05-169).

Of the eight species of insect tested, five species, including the cowpea aphid (*Ap. craccivora*), diamondback moth (*Pl. xylostella*), striped flea beetle (*Ph. striolata*), silverleaf whitefly (*Be. argentifolii*) and oriental fruit fly (*Ba. dorsalis*), were susceptible and infected by both the *M. anisopliae* transformant (MA05-169) and the wild type (BCRC35505). In contrast, the cabbage worm (*Pi. rapae*), rhinoceros beetle (*Al. dithotomus*), and tobacco cutworm (*S. litura*) were not susceptible (Table 2). Thus, the transformant and wild-type strains could both infect species representative of several insect orders, i.e., Lepidoptera, Hemiptera, Diptera, Coleoptera, and Homoptera. The transformant was transformed and inserted with two copies each of the polyketide synthase, scytalone dehydratase, and 1,3,8-trihydroxynaphthalene reductase genes [5]. The presence of these six copies of genes in did not change the insect host specificity of the transformant. However, out of the three Lepidoptera tested, only *Pl. xylostella* was susceptible and infected. Likewise, out of the two Coleoptera tested, only the striped flea beetle (*Ph. striolata*) was susceptible and infected. Although this outcome suggests that both strains have the same host range, they still exhibited a certain level of host preference (Table 2). As shown in a previous study, the transformant caused the infected *Pl. xylostella* to display black patches and brown-blackish droplets of exudation on its cuticle [5]. Similarly, blackish droplets also occurred on the *Ap. craccivora* at a later stage of infection (Fig. 1C). Although the contents of the brown-blackish droplets were still not known, they might contain melanin or an allied compound, orthosporin, which was discovered in this study (Figs. 2, 3). Both the transformant and wild type were able to infect the adult striped flea beetle, *Ba. dorsalis*, and the *Be. argentifolii* nymph, though the former two sporulated less profusely than the latter, while still being capable of completing the disease cycle (Fig. 1). In particular, it is worth noting that the sporulation of both the transformant and wild type were obviously less profuse on the *Ap. craccivora*, indicating that the sporulation capacity can change drastically depending on the host-parasite interaction.

**Table 2:** The host range of *Metarhizium anisopliae* transformant (MA05-169) and wild type (BCRC35505).

Order	Insect	Instar	MA05-169	BCRC35505*
Family				
Lepidoptera				
Noctuidae	Tobacco cutworm (*Spodoptera litura*)	2nd	−	−
*Pieridae*	Cabbage worm (*Pieris rapae*)	2nd	−	−
Plutellidae	Diamondback moth (*Plutella xylostella*)	2nd	+	+
Coleoptera				
Chrysomelidae	Striped flea beetle (*Phyllotreta striolata*)	Adult	+	+
Scarabaeidae	Rhinoceros beetle (*Allomyrina dithotomus*)	2nd∼3rd	−	−
Hemiptera				
Aleyrodidae	Silverleaf whitefly (*Bemisia argentifolii*)	2nd∼3rd	+	+
Aphididae	Cowpea aphid (*Aphis craccivora*)	2nd∼3rd	+	+
Diptera				
Tephritidae	Oriental fruit fly (*Bactrocera dorsalis*)	Adult	+	+

*+, infected; −, non-infected.

### Conidial germination and appressorial differentiation *in vitro* and *in vivo*

**Figure 4:**
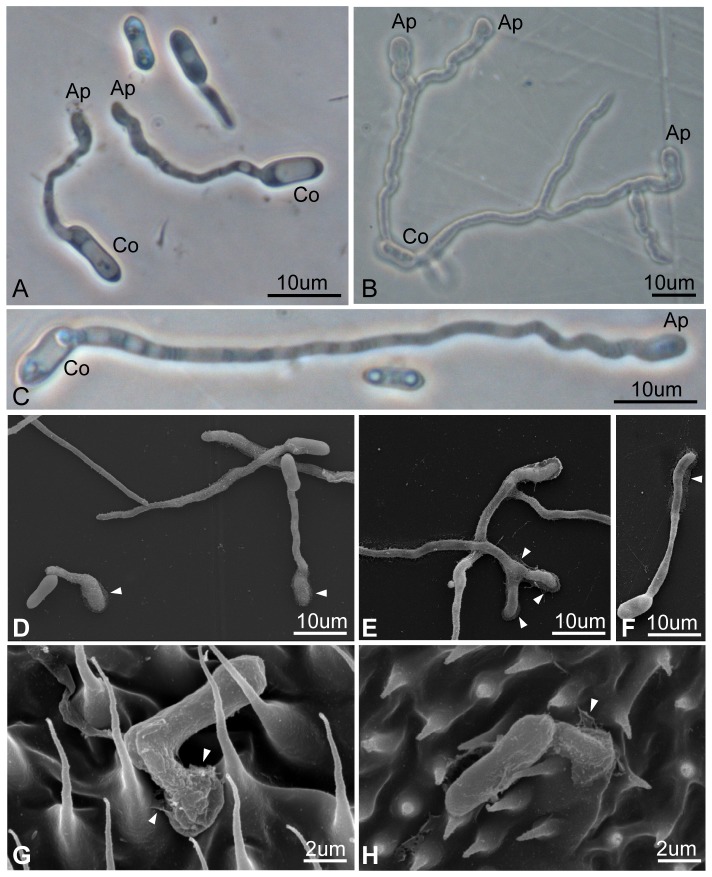
Light and scanning electron microscopy of conidia germination and formation of the appressorium of *Metarhizium anisopliae* transformant (MA05-169) and wild type (BCRC35505) on a mylar polyester film or on a diamondback moth (*Plutella xylostella*). A–B) MA05-169, 16 and 48 h after spreading onto a mylar polyester film for 16 and 48 h, respectively; C) BCRC35505, 72 h after spreading. D–E) Note the presence of a distinct extracellular adhesion mucilage on the appressorium of the transformant after germination for 16 h and 72 h on the mylar polyester film (white arrow heads) compared with the indistinct appressorium and barely visible mucilage of the wild type at 72 h (arrow head) (F). G) The growth of the transformant on the insect body surface after inoculation for 16 h compared with the growth of the wild type for 24 h (H). Co: conidia; Ap: appressorium.

The *M. anisopliae* transformant germinated considerably faster and more vigorously than the wild type strain, as determined by the *in vitro* assay on a mylar polyester film. For instance, after incubation for 16–24 h, the conidial germination rate of the transformant compared to the wild type had a ratio of 81.03∼87.26% compared to 4.18∼12.48%. The sharp distinction between them ranged from 19.4- to 7-fold (Table 3). Even after 64 h, the conidial germination rate of the wild type was still less than 20% (Table 3). The conidia of the transformant not only germinated faster but also branched more and produced many more appressoria compared with the wild type. The difference between them exceeded 80-fold at 24 h (Table 3; Figs. 4 A to 4C). Additionally, under scanning electron microscopy, the mucilage covering the appressorium of the transformant appeared much more prominent than that in the wild type (Figs. 4D to 4F). This same behavior was also observed on the infected *Pl. xylostella* (Figs. 4G and 4H).

**Table 3:** The rate of germination and appressorium formation of *Metarhizium anisopliae* transformant (MA05-169) and wild type (BCRC35505) on a mylar polyester film.

Incubation time (h)	MA05-169*				BCRC35505*			
	GR (%)	±SE	AR(%)	±SE	GR (%)	±SE	AR (%)	±SE
16	81.03a	2.39	60.71A	2.46	4.18b	2.15	0.00B	0.00
20	83.60a	0.46	72.13A	3.08	3.98b	0.3	0.65B	0.65
24	87.26a	0.05	80.21A	1.84	12.48b	2.57	0.00B	0.00
48	NA		NA		19.33	4.16	1.28	1.28
64	NA		NA		19.74	1.83	4.16	2.19

*The data of germination rate (GR) and appressorium formation rate (AR) were analyzed separately by one-sample *t*-test at *P*<0.05. Values with different letters were significantly different from each other. SE: standard error; NA: not applicable.

**Figure 5:**
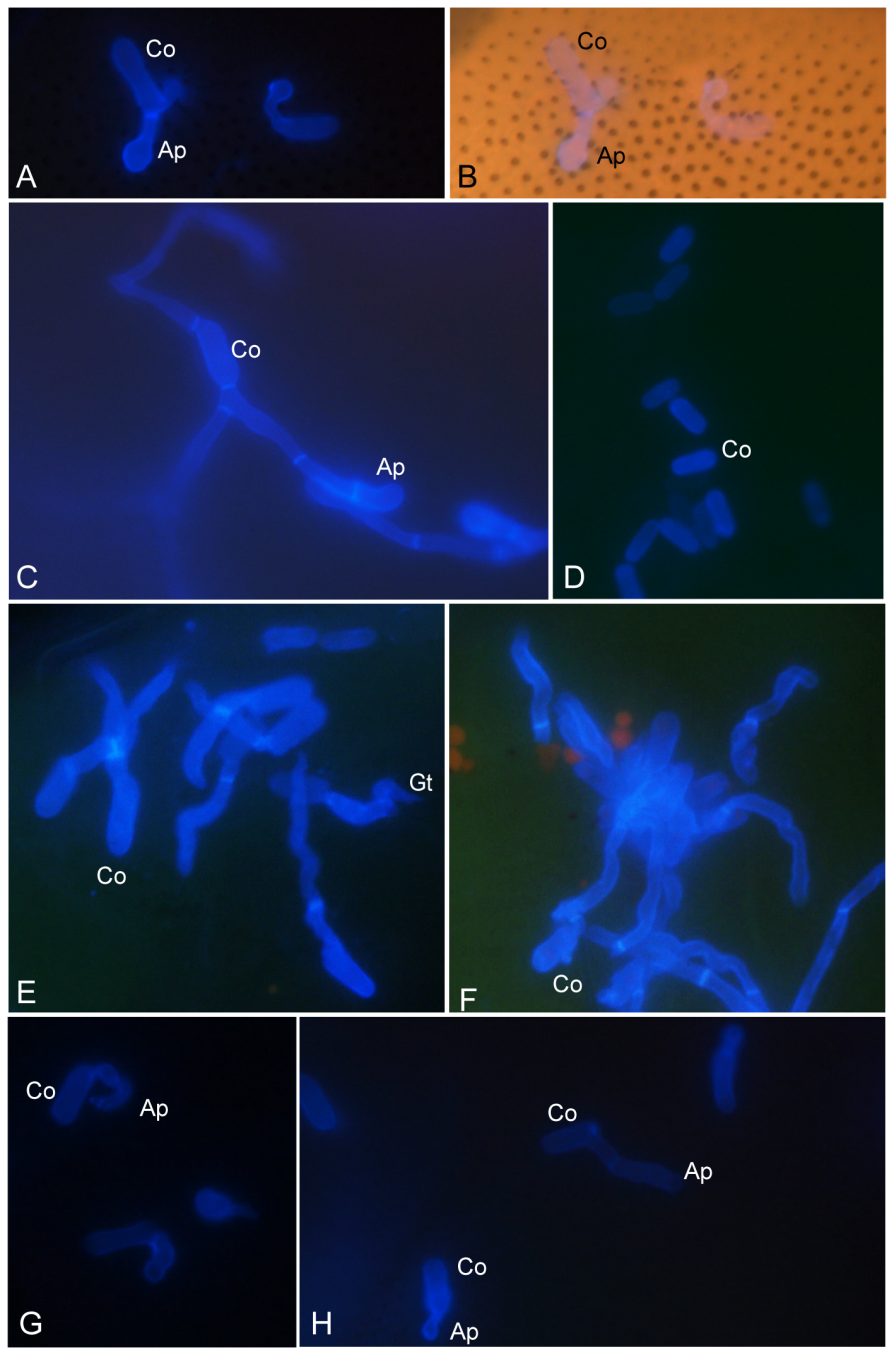
Light and fluorescent microscopy of conidia germination and formation of appressorium of *Metarhizium anisopliae* transformant (MA05-169) and wild type (BCRC35505) on different insect hosts. A–B) MA05-169 on the diamondback moth (*Plutella xylostella*); B, merged visible light and UV-fluorescent images; C, E, G) MA05-169 on the striped flea beetle (*Phyllotreta striolata*), cowpea aphid (*Aphis craccivora*), and oriental fruit fly (*Bactrocera dorsalis*), respectively; D, F, H) BCRC35505 on the *Ph. striolata*, *Ap. craccivora*, and *Ba. dorsalis*, respectively.

In the *in vivo* analysis of four different insect hosts, the rate of conidial germination and appressorium formation of the transformant and wild type varied, depending on the insect host infected (Table 4). On *Ap. craccivora*, both strains had the highest germination rate in the range of 82.5 to 87.5% and the lowest appressorium differentiation rate in the range of 5.1 to 6.0%, suggesting the possibility of direct host penetration by the germ tube. In addition, the differences in the rate of germination and appressorium differentiation in the transformant and wild type were not significant (Table 4; Figs. 5E and 5F). Notably, on *Ph. striolata*, the transformant displayed the 2nd highest germination rate (GR) (66.3%) and appressorium formation rate (AR) (51.4%), which is in significant contrast to the rates of the wild type (GR 5.7%; AR 7.5%), with an 11.6-fold (GR) and 6.9-fold (AR) difference, respectively (Table 3; Figs. 5C and 5D). For the infected oriental fruit fly (*Ba. dorsalis*), the rate of conidial germination and appressorium formation in the transformant versus the wild type was 48.4∶51.8% (GR) and 26.0∶22.6% (AR), respectively, but these ratios were not statistically significant (Table 4; Figs. 5G and 5H). With respect to the infected *Pl. xylostella*, a sharp distinction regarding the GR (39.8% vs. 12.7%) and AR (67.2% vs. 25.8%) of the transformant and wild type was also observed (Table 4).

**Table 4:** The rate of germination and appressorium formation of *Metarhizium anisopliae* transformant (MA05-169) and wild type (BCRC35505) on different insect hosts.

Insect	MA05-169*				BCRC35505*			
	GR (%)	±SE	AR (%)	±SE	GR (%)	±SE	AR (%)	±SE
*Aphis craccivora*	87.5a	4.0	5.1A	1.3	82.5a	5.8	6.0A	2.7
*Bactrocera dorsalis*	51.8a	2.6	26.0A	2.9	48.4a	1.6	22.6A	3.5
*Phyllotreta striolata*	66.3a	2.2	51.4A	4.3	5.7b	1.6	7.5B	3.8
*Plutella xylostella*	39.8a	4.0	67.2A	5.5	12.7b	3.1	25.8B	3.8

*The data of germination rate (GR) and appressorium formation rate (AR) were analyzed separately using a one-sample *t*-test with *P*<0.05. Values with different letters were significantly different from each other. SE: standard error.

### Appressorium turgor pressure

There was no significant difference in the appressorial turgor pressure of the transformant (MA 05-169) and the wild type (BCRC 35505) differentiated on cricket hind wings, as shown by the rate of collapsed appressoria. The collapse of the appressoria became evident after the cricket wings were immersed in a higher concentration of PEG-8000 solution for 10 min, which was linearly proportional to the concentration of PEG-8000. The appressorium collapse rate reached approximately 65% when the cricket wing was immersed in PEG-8000 at a concentration of 1.2 g ml^−1^. This outcome suggests that the DHN-melanin in the transformant did not act as an adequate osmoticum to force the influx of additional free water into appressoria and thus could not increase the turgor pressure sufficiently to counteract the osmotic pressure generated by the high PEG-8000 concentration.

### Tolerance of the transformant and wild type to H_2_O_2_ and nitric oxide stress

**Figure 6:**
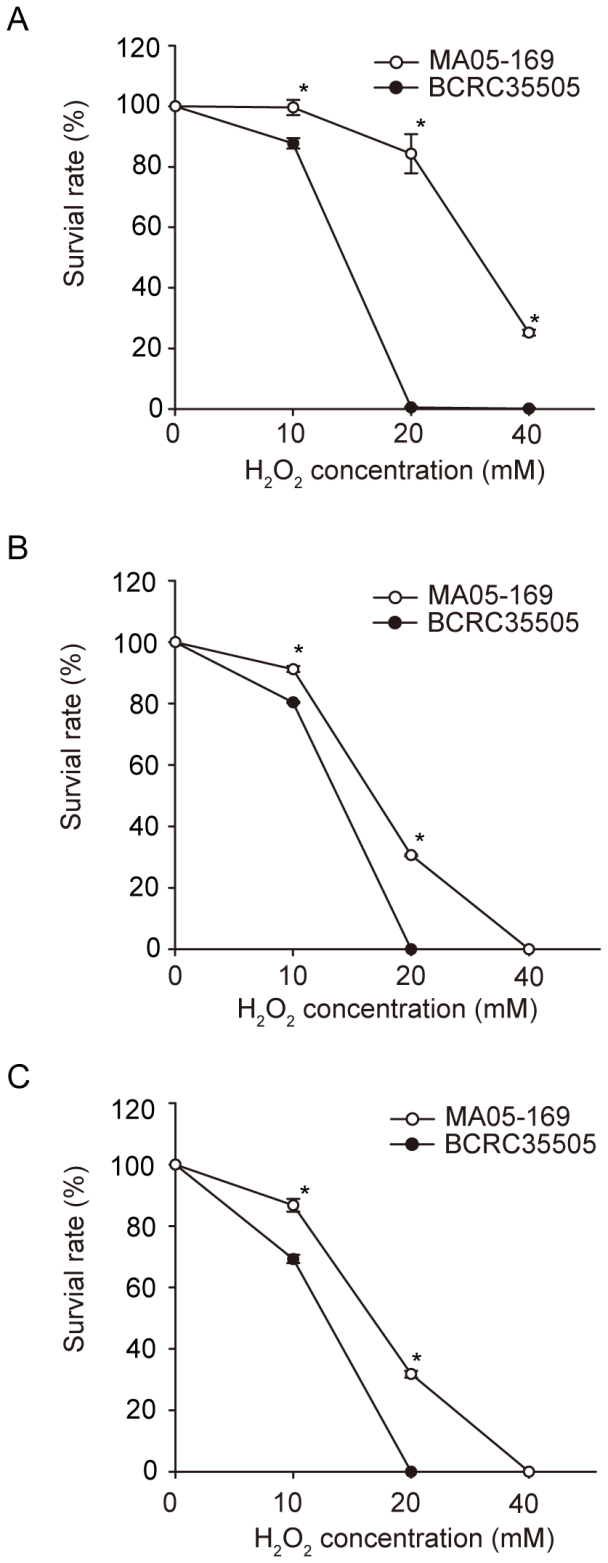
Conidia survival rate of *Metarhizium anisopliae* transformant (MA05-169) and wild type (BCRC35505) treated with different concentrations of hydrogen peroxide (H_2_O_2_) for 20 (A), 40 (B), or 60 (C) min, respectively. The error bars indicate the standard deviations. Points denoted with an (*) represent significant differences (*P*<0.05).

**Figure 7:**
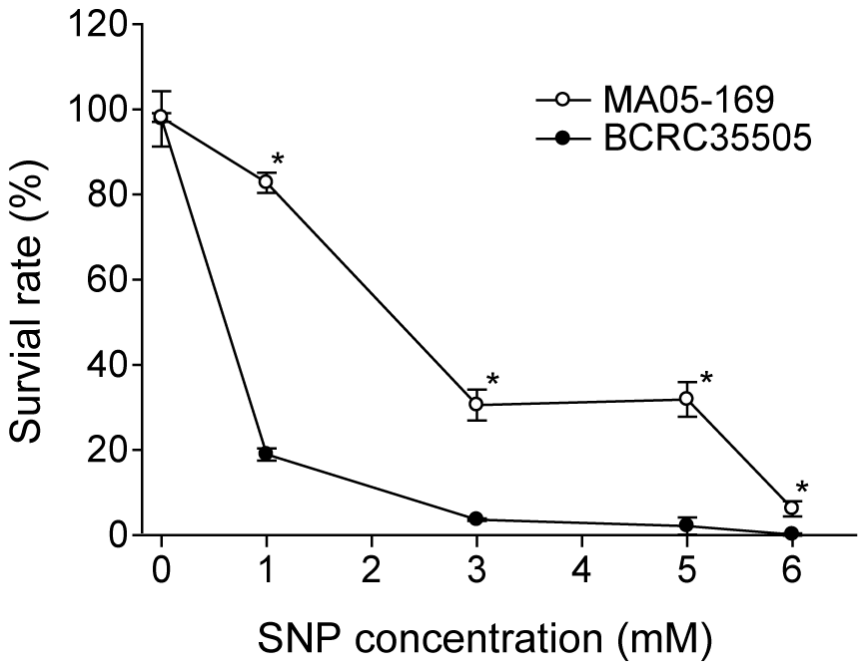
The difference in the conidia germination rate of *Metarhizium anisopliae* transformant (MA05-169) and wild type (BCRC35505) after treatment with sodium nitroprusside (SNP) for 16 h. The error bars indicate the standard deviations. Points denoted with an (*) represent significant differences (*P*<0.05).

In general, the rate of conidial survival of the transformant was much higher than that of the wild type, following treatment with either H_2_O_2_ or sodium nitroprusside (SNP) (Figs. 6, 7). However, the survival rate of the transformant and wild type declined in proportion to the increase in the H_2_O_2_ concentration and the treatment time interval, though the decline slope of the transformant was less steep than that of the wild type (Figs. 6A to 6C). This indicated that the transformant was more tolerant to the detrimental effects of H_2_O_2_ (Figs. 6A to 6C). For example, after treatment with 10 mM H_2_O_2_ for 20 min, the transformant possessed an almost 100% survival rate. In contrast, the survival rate of the wild type was approximately 85%. At 20 mM, the survival rates were 80% for the transformant and 0% for the wild type (Fig. 6A). Likewise, after being treated with 10 mM H_2_O_2_ for 40 min, the transformant had a survival rate of 90%, while the wild type had a survival rate of 80%. At 20 mM, the survival rate of the transformant was 25% and that of the wild type was 0% (Fig. 6B). The same trend was observed during treatment with H_2_O_2_ for 60 min at 10 mM: the survival rate of the transformant was approximately 85%, whereas the survival rate of the wild type was 65%. At 20 mM, the survival rate of the transformant was 20% and that of the wild type was 0% (Fig. 6C). After treatment with 1 mM SNP for 16 h, 80% of the transformant population survived, while only 20% of the wild type population survived. At 2 mM SNP, approximately 25% of the transformant survived, but only 5% of the wild type survived. Finally, at 5 mM SNP, the survival rate of the transformant was 25% and that of the wild type was 0% (Fig. 7).

### Production of destruxin and allied compounds in transformant and wild type *M. anisopliae*

Crude extract of culture filtrates from the transformant or wild type were partially purified by acetate and methanol. The resulting residue was diluted one hundred-fold and assayed using HPLC. The cytochalasin of C and D were absent and undetectable in both strains. The quantity of destruxin A in the transformant and wild type could be determined using a standard calibration curve based on the authentic destruxin A purchased from Sigma (USA). The retention time of the destruxin A was 32.8 min (Fig. 2). The concentration of destruxin A in the wild type was 0.174 mg/ml, and while this was a very small amount, it was still 3.2-fold greater than that of the transformant (0.054 mg/ml). In contrast to the wild type, an unknown compound represented by a high peak eluting at 31.11 min was produced by the transformant. This unknown peak was 43.7 times higher in the transformant compared to the wild type (Fig. 2). The compound corresponding to this peak was further purified to homogeneity and subjected to analysis by LC/MS and NMR spectroscopy. The compound was identified as orthosporin (Figs. 2, 3), which has been identified and demonstrated previously with anti-microbes, -insects, and -cancer cells capacity [39], [40].

### Enhanced expression of cuticle hydrolytic enzymes encoding genes

Previous studies have demonstrated the crucial roles of extracellular chitinase, protease, and phospholipase in the infection and colonization of insect hosts [33], [35], [36]. We developed absolute quantitative PCR (AQ-PCR) to determine the expression levels of these three enzymes encoding genes in the transformant and wild type. *In vitro*, preliminary investigations based on the extraction of genetic material from mycelium showed that the transcript levels of chitinase *chit l*, protease *pr1D*, and *phospholipase C* in the melanized transformant were all significantly higher than in the wild type (data not shown).

*In vivo*, the expression of these three genes in the *Pl. xylostella* after inoculation for 16, 24, 48, and 96 h was examined using AQ-PCR. Initially, at 16 h, the expression of these three genes in the transformant or wild type did not show significant difference. However, after 24 h, the expression of chitinase *chit 1*, protease *pr1D*, and *phospholipase C* in the transformant were all enhanced considerably, at ratios of 7, 14, and 61 times compared with the wild type (Table 5). At a later infection stage at 48 h, the enhanced overexpression of these three genes was even more pronounced in the transformant compared with the wild type; the former expressed 67.7-, 100-, and 312.5-fold higher copy transcript numbers than the latter (Table 5). At 96 h, the differential expression of these three genes between the transformant and wild type was diminished, although 10-, 19-, and 11-fold differences were still observed (Table 5). Coincidentally, the overexpression of pathogenicity-relevant genes in the transformant compared with the wild type was also demonstrated by a transcriptomic study (data not shown).

**Table 5:** The differentially expressed transcript of protease *pr1D*, chitinase *cht1*, and phospholipase C genes of *Metarhizium anisopliae* transformant (MA05-169) and wild type (BCRC35505) on the *Plutella xylostella* after inoculation for 24, 48, and 96 h.

Genes	Transcript copy no.^a^					
	MA05-169			BCRC35505		
	24 h	48 h	96 h	24 h	48 h	96 h
Chitinase *chit1*	83	2.1×10^3^	7.1×10^3^	12	31	0.7×10^3^
*Phospholipase C*	14	0.7×10^3^	11.6×10^3^	NA	7	0.6×10^3^
Protease *pr1D*	61	5.0×10^3^	12.7×10^3^	NA	16	1.2×10^3^

a NA, not applicable.

### Effect of melanin biosynthesis inhibition on the median lethal time (LT_50_) of the insect hosts

We inhibited the function of two DHN-melanin biosynthesis enzymes, tri- and tetrahydroxynaphthalene reductase, in the transformant with tricyclazole to prove the critical role played by the melanin [31], [41], [42]. In the tricyclazole-treated transformant, the median lethal time (LT_50_) for the *Pl. xylostella* was extended from 47.6 h to 54.9 h; this time interval was still shorter than that for the wild type, although the difference was not statistically significant (Table 6). By contrast, the LT_50_ for the transformant without tricyclazole treatment was significantly shorter than the LT_50_ for wild type on *Pl. xylostella* (47.66 h vs. 56.78 h) (Table 6).

**Table 6:** The median lethal time (LT_50_) for the *Plutella xylostella* infected by spores of *Metarhizium anisopliae* transformant (MA05-169) and wild type (BCRC35505) treated with or without tricyclazole.

Lethal time and confidence^a^	Conidia tricyclazole (TCA) treatment^b^			
	MA05-169		BCRC35505	
	w/TCA	wo/TCA	w/TCA	wo/TCA
LT_50_ (hours)	54.90	47.66	58.82	56.78
95% Confidence limits (lower-upper)	57.8–58.3	44.4–51.0	54.4–62.7	53.9–60.0

a Median lethal time (LT_50_) was determined by Probit regression analyses with a 95% significance limit, with (w) or without (wo).

b TCA treatment.

Likewise, in additional tests, except for *Ap. craccivora* and *Ba. Dorsalis*, *Ph. striolata* showed a significantly shorter LT_50_ when infected by the transformant compared to the wild type (Table 7). It was noticed that the body of the *Ap. craccivora* was fleshy, soft, and fragile and was thus easily penetrated by the undifferentiated germ tube of either the transformant or the wild type (Figs. 5E and 5F).

**Table 7:** Median lethal time (LT_50_) for the cowpea aphid (*Aphis craccivora*), orient fruit fly (*Bactrocera dorsalis*), and striped flea beetle (*Phyllotreta striolata*) infected by *Metarhizium anisopliae* transformant (MA05-169) and wild type (BCRC 35505).

Lethal time and confidence^a^	*Aphis craccivora*		*Bactrocera dorsalis*		*Phyllotreta striolata*	
	T^b^	W	T	W	T	W
LT_50_ (hours)	48.0	50.6	80.4	86.5	82.4	100.2
95% Confidence limits (lower-upper)	41.0–60.1	43.2–63.8	58.9–108.7	61.9–122.8	76.2–90.5	92.3–114.9

a Median lethal time (LT_50_) was determined by Probit regression analyses with a 95% significance limit.

b T, transformant MA05-169; W, wild-type BCRC35505.

### Colonization and survival of transformant and wild type *M. anisopliae* on the rhizoplane

**Figure 8:**
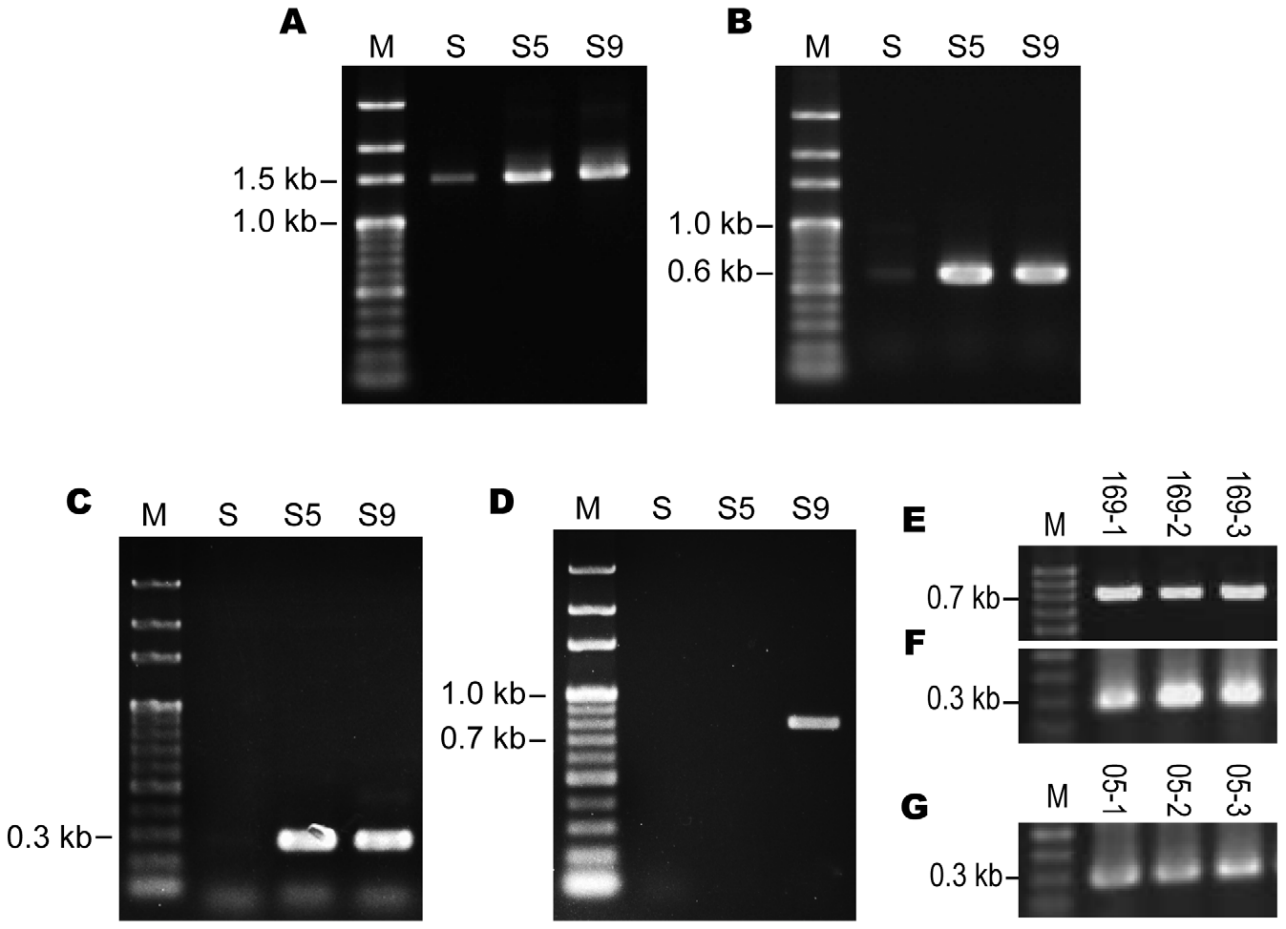
Detection of the colonization capacity of *Metarhizium anisopliae* wild type (BCRC 35505) and transformant (MA05-169) on cabbage rhizoplane and in rhizosphere by direct or nested polymerase chain reaction (PCR) using 16S, ITS, IGS or PKS primers. A) An extremely fuzz *16S* band, implicating the presence of soil inhabiting microbes (S), is in contrast to the distinct 16S band of the *M. anisopliae* wild type (S5) and transformant (S9). B–D) Neither *ITS* (B), *IGS* (C) nor *PKS* (D) are detectable in the rhizosphere (S). By contrast, both *ITS* (C) and *IGS* (D) are present in the wild type (S5) and transformant (S9), whereas *PKS* is only present in the transformant (S9) (D). E–G) Detection of the transformant from isolates from the inoculated roots generated with specific primers PKS_TE(a) and PKS_TE(b) by direct PCR (E); and using primers Ma-28S4, Ma-IGS1, and Ma-IGSspF, MaIGSspR for two-steps nested PCR for the detection of transformant (F) and wild type (G). M: 100 bp ladder; S: DNA extracted from loam soil; S5: DNA extracted from loam soil combined with a spore suspension of BCRC 35505; S9: DNA extracted from loam soil combined with spore suspension of MA05-169; 169-1 to 169-3, and 05-1 to 05-3 represent isolates of the transformant and wild type, respectively.

In the rhizoplane colonization test, we conducted PCR to determine whether the loam soil was free of *M. anisopliae* and whether the inoculation was successful. Sets of specific primers targeting 16S *rDNA*, *ITS*, *IGS*, and *PKS* were used. In the loam soil sample (Figs. 8A to 8D, lane “S”), except for the presence of soil-inhabiting microbes detected by 16S *rDNA* and *ITS* primers, no *M. anisopliae* signal could be detected. This indicated that the soil used for growing the cabbage seedlings did not contain *M. anisopliae* propagules. In Fig. 8C, the specific *IGS* band can only be visualized in wild type and transformant *M. anisopliae* (lanes “S5” and “S9”), indicating the specificity of the two-step nested PCR method for detecting *M. anisopliae* [37]. The specificity of the *PKS* primers for detecting the transformant was also confirmed, as a single *PKS* band of approximately 700 bp was only observed in the transformant (Fig. 8D, lane “S9”).

In the DNA extracted from the culture of *M. anisopliae* MA05-169 and BCRC35505 from the rhizoplane of inoculated cabbage seedlings at 7, 14, 21, 28, and 35 days, a specific band at approximately 300 bp was produced (Figs. 8 F and 8G) with IGS primers Ma28S4, Ma-IGS1 and Ma-IGSspF, MaIGSspR in a two-step nested PCR. In the seedlings inoculated with the *M. anisopliae* MA05-169 conidial suspension, a specific signal at approximately 700 bp could be detected using primers PKS_TE (a) and PKS_TE (b) (Fig. 8E) [5]. These results indicate that both *M. anisopliae* MA05-169 and BCRC 35505 could colonize the rhizoplane of cabbage seedlings and survive even at 35 days after inoculation (Figs. 8D to 8G).

## Discussion

### Non-relevance of melanin biosynthesis to the build-up of appressorial turgor pressure in the *M. anisopliae* transformant MA05-169

Melanin can be deposited between the inner side of the appressorial cell wall and cell membrane, forming a semipermeable dense layer. Because the melanin layer is only permeable to molecules equal to or smaller in size than water, a massive turgor pressure can accumulate in the appressorium, facilitating the penetration of the host epidermal cells. While the melanin layer has been recognized as an important virulence factor in a diverse range of plant and human pathogenic fungi [29], [43]–[45], no study has investigated the association of melanin and appressorial turgor pressure in entomopathogenic fungi, even though appressorial turgor pressure is known to be crucial for their virulence [46], [47].

In this study, no significant difference in the appressorial turgor pressure was observed between the *Metarhizium anisopliae* transformant MA05-169 and the wild type BCRC35505, suggesting that melanin biosynthesis in MA05-169 does not lead to a build-up of turgor pressure in the appressorium. We speculate that it might be attributed to melanin dispersion rather than aggregation in the appressorial cell. Thus far, the mechanisms controlling the distribution pattern and deposition of melanin in fungal cells have remained unclear. Previous studies have shown that in *Colletotrichum lagenarium*, the deletion of *cmr1* (*Colletotrichum* melanin regulation) affects melanin production in the mycelium, but not in the appressorium [48]. Two genes encoding the transcription factors of melanin biosynthesis in *Magnaporthe grisea* and *Bipolaris oryzae*, *Pig1* (pigment of *Magnaporthe*) and *BMR1* (*Bipolaris* melanin regulation), have been characterized [48], [49]. In *Sordaria macrospora*, melanin biosynthesis and the expression of four genes involved in the pathway only occurred during the development of perithecia [50]. The above evidence suggests the existence of fine-tuned systems for regulating the biosynthesis, delivery, and/or deposition of DHN-melanin in fungal cells. It is likely that in *M. anisopliae* MA05-169, where the complex modulation system may be missing, the synthesized DHN-melanin cannot be directed to accumulate at the appressorial cell wall. The lack of a specialized melanin layer therefore caused no changes in the osmotic potential or turgor pressure in the appressorium of the melanized transformant.

### Melanin acts as a key virulence factor in the *M. anisopliae* MA05-169 transformant

The association between melanin and virulence has been reported in a wide range of fungal pathogens, such as *Cryptococcus neoformans*, *Aspergillus fumigatus*, and *Paracoccidioides brasiliensis*. It is known that for many pathogenic animal fungi, melanin functions during pathogenesis as a scavenger to eliminate free radicals generated by the host immune system [29]–[31], [42]. To understand the effect of melanin in *M. anisopliae* MA05-169, the transformant was tested for tolerance against H_2_O_2_- or NO-mediated free radicals *in vitro* and *in vivo* for the alteration in virulence after treatment with a melanin biosynthesis inhibitor (tricyclazole).

Melanin is characterized by the presence of stable unpaired electrons in the polymer, which come from trapped free radicals [51]. Our previous study showed that the melanin compound purified from *M. anisopliae* MA05-169 also contained stable unpaired electrons [5]. In this research, after exposure to the nascent oxygen atom or nitric oxide derived from H_2_O_2_ or SNP, respectively, the *M. anisopliae* MA05-169 conidia exhibited a significantly greater survival rate than the wild-type conidia, indicating that the exogenous melanin in the transformant may be effective in trapping and eliminating free radicals in the environment. The protective effect of melanin has been commonly observed in many organisms. For example, in *C. neoformans*, the melanized cells were more resistant against nitrogen- and oxygen-derived oxidants than the nonmelanized cells [52].

Tricyclazole, an effective inhibitor of hydroxynaphthalene (HN) reductase that is involved in the dihydroxynaphthalene (DHN) melanin biosynthesis pathway, has been widely used to help demonstrate the presence and roles of DHN-melanin in fungi. Studies have explored the association of melanin and appressorial turgor pressure in plant pathogenic fungi [34], [53] and investigated how animal pathogenic fungi utilize melanin against the host immune system [54], [55]. In this research, the enhanced virulence of the melanized *M. anisopliae* transformant MA05-169 in *Plutella xylostella* larvae was compromised by a treatment with tricyclazole, indicating a positive correlation between the melanin production and the increased virulence. While tricyclazole is a recognized systemic fungicide that can be absorbed into and translocated within plant tissues [56], whether tricyclazole is taken up into the insect bodies is unknown. Because the *M. anisopliae* spores were suspended in a tricyclazole solution for inoculation, tricyclazole should be able to make direct contact with not only the spores but also the germ tubes and appressoria on the insect surface, regardless of its ability to permeate the insect body. The primary infection hyphae growing from the appressorium might also be indirectly affected. These most likely led to the suppression and/or delay of melanin biosynthesis during the early stages of pathogenesis.

The results of our *in vitro* and *in vivo* tests suggest that melanin is likely a key virulence factor in the *M. anisopliae* transformant MA05-169. From the early stages of pathogenesis, melanin eliminated free radicals such as ^ ˙^O_2_^−^ that were generated by the host immune system, giving the melanized *M. anisopliae* transformant MA05-169 an effective head start in infection and colonization. In contrast, with the treatment of tricyclazole, melanin biosynthesis in *M. anisopliae* MA05-169 was suppressed and/or delayed, such that it no longer had the advantage of being protected from free radical damage. As a result, the tricyclazole-treated *M. anisopliae* MA05-169 was less virulent than the untreated *M. anisopliae* MA05-169.

### Correlation of enhanced germination and appressorium formation abilities with the increased virulence of the *M. anisopliae* transformant MA05-169

For many entomopathogenic fungi, pathogenic development is initiated from conidial germination and appressorium formation, followed by the secretion of adhesive substances that affix the appressorium to a host insect's body surface [57]. This enables the invasive hyphae to penetrate the insect cuticle. Microscopic investigations were performed in this study to examine the *M. anisopliae* transformant MA05-169 and the wild type at the pre-penetration stage. When placed on either the mylar membrane or on *Pl. xylostella*, both strains developed inflated and irregular-shaped or elliptical appressoria at the ends of their germ tubes. The morphological phenotypes of the appressoria and the adhesive substances visible below the base of the appressoria were consistent with those observed by Arruda et al. (2005) during the interaction between *Metarhizium anisopliae* and *Boophilus microplus* [57]. This indicates that transgenesis does not impair the appressorium function of *M. anisopliae* MA05-169.

Our previous study has shown that when cultured on PDA medium, the germination and hyphal growth abilities of the *M. anisopliae* transformant MA05-169 were superior to those of the wild type [5]. In this study, we further revealed that compared with the wild type, *M. anisopliae* MA05-169 showed much greater efficiencies of conidial germination and appressorium formation both *in vitro* (on the mylar membrane) and *in vivo* (on *Phyllotreta striolata* and *Plutella xylostella*, but not on *Aphis craccivora* or *Bactrocera dorsalis*). Because germination and appressorium formation are essential for the infection of many entomopathogenic fungi, it is reasonable to infer that the higher virulence of *M. anisopliae* MA05-169 could be at least partially explained by its enhanced germination and appressorium formation abilities, although the underlying cause of such an enhancement is unclear. Currently, we do know that melanin disperses in the cytoplasm, as visualized under confocal scanning light microscopy (data not shown). However, the exact ultrastructural distribution of melanin in the spore or appressorium of the transformants remains unknown. A study comparing the infection model of both the transformant and wild type on *Pl. xylostella* is underway, and this study should resolve the puzzle. In other aspects, the hydrophobic attraction force and water absorption capacity of the melanized transformant should be enhanced, which in turn should facilitate and consolidate the adhesion of the hydrophobic conidia to hydrophobic insect host cuticle, thereby enhancing the water uptake from the surrounding atmosphere. The physical or chemical cues on the insect cuticle will further stimulate the germination and formation of the appressorium and the secretion of mucilages and hydrolytic enzymes (chitinase, phospholipase and protease, etc.) [58]–[60]. In combination, these factors might help to account for the enhanced virulence of the transformant compared with the wild type. It is also possible that the global metabolism and physiological functions of *M. anisopliae* MA05-169 have been affected due to the introduced exogenous genes and/or the alterations in the expression of some endogenous genes located across from or near the T-DNA insertion sites. However, the NGS and BLAST analyses of the available sequences encompassed in the eight fosmid clones did not allow us to further identify genes likely involved in fungal germination/appressorium formation.

Another interesting observation is that there are differential levels of virulence of *M. anisopliae* (wild type or transformed) in various insect hosts. It is also worth noting that the transformant MA05-169 exhibited elevated levels of virulence in some but not all of the insect hosts being tested in this study. Differences in virulence-associated phenotypes such as germination rate and appressorium formation suggest the presence of some determining factors in the insect hosts' body walls. The insect cuticle is a dynamic structure, and thus, the lipid acids and wax precursors secreted by epidermal cells can be transported to the surface through the pore canal system [61]. The components of the insect cuticle can induce spore germination and allow certain fungi to enter [62]. The cuticle also contains substances/compounds that are selectively inhibitory to particular fungi. For instance, caprylic acid, valeric acid, and nonanoic acid on the larval surface of corn earworms and fall armyworms possessed mycostatic activity toward *Beauveria bassiana* [63]. Barnes & Moore (1997) have reported that short-chain fatty acids (such as caprylic acid) inhibit the germination of *Metarhizium flavoviride* spores [64], whereas long-chain fatty acids promote their germination. James et al. (2003) also indicated that cuticular lipids extracted from silverleaf whitefly nymphs (*Be. argentifolii*) affect the conidial germination of *Beauveria bassiana* and *Paecilomyces fumosoroseus* [65]. Poor germination behavior of *M. anisopliae* was observed on the surface of live but not dead flea beetle cuticles, indicating that the body wall of a live flea beetle may contain a substance or substances that can inhibit *M. anisopliae* germination [66]. In entomopathogenic fungi, appressorium differentiation can also be affected by the cuticular structures of different insects. For both the transformant MA05-169 and the wild type, the *in vivo* germination rates were found to be high (>80%) on *A. craccivora*; however, less than 5% of the germinated spores formed appressoria. Similar findings were observed during the infection of *Myzus persicae* and *Lipaphis erysimi* by *M. anisopliae* [66]. It is likely that the appressorium is not a required infection structure for *M. anisopliae* to penetrate the soft-bodied *Ap. craccivora*. Instead, the infection of *Ap. craccivora* mainly occurs through the direct penetration of infectious hyphae.

### Secondary metabolites produced by transformant and wild type *M. anisopliae*

To understand whether and how the expression of exogenous *PKS*, *SCD*, and *THR* genes in *M. anisopliae* MA05-169 changes its overall metabolism, *in vitro* metabolic profiles were investigated using HPLC, LC/MS, and NMR analyses. Two differentially produced compounds were identified in the study – the transformant MA05-169 accumulated 3.2 times less destruxin A and 43.7 times more orthosporin than the wild type. Orthosporin is a polyketides [67], a large class of secondary metabolites that are biosynthesized through the involvement of diverse *PKS* genes. Although the *in vitro* conditions do not fully reflect *in vivo* mechanisms, changes in melanin and orthosporin levels indicated that the exogenous genes (*PKS* in particular) may have altered the complex polyketide biosynthesis network in *M. anisopliae*. This hypothesis was further supported by the whole-transcriptome RNAseq analyses of the transformant and the wild type grown in liquid SDB cultures (data not shown). Through the transcriptome analysis, six endogenous *PKS*-like genes were found to be over- or under-expressed by more than twofold in *M. anisopliae* MA05-169.

For the commercially available mycotoxins we chose to test, a significant difference between the transformant and the wild type was only observed in destruxin A production. Destruxin A is a cyclic hexadepsipeptide that is produced by several taxonomically distinct fungi, in which destruxin A has a wide range of biological activities against their respective hosts [68]. In the *Metarhizium* spp. biosynthetic pathway of destruxins, the nonribosomal peptide synthetase DtxS2 is responsible for the conversion of destruxin B into destruxin C, destruxin D, and then destruxin A [69]. Of the six differentially expressed genes identified from the transcriptome analysis, a putative *dtxS2* gene was found to be downregulated, which is consistent with the finding of a reduced level of destruxin A in *M. anisopliae* MA05-169 (data not shown). In spite of some previous evidence regarding the role of destruxin A in *M. anisopliae* pathogenicity [70], [71], the suppression of destruxin A in *M. anisopliae* MA05-169 suggests that it may not account for the enhanced virulence of *M. anisopliae* MA05-169.

Orthosporin (de-O-methyldiaporthin) is an isocoumarins that is produced by *Drechslera siccans*, *Aspergillus ochraceus*, *Ulocladium* sp., and *Daldinia concentrica* [67], [72]–[74]. This compound had not been previously identified in *M. anisopliae* or other entomopathogenic fungi [73], [74]. Our HPLC and NMR analysis detected a dramatic increase in orthosporin production in the *M. anisopliae* transformant MA05-169, indicating the activation of an unknown biosynthetic pathway to produce a novel metabolite. A similar phenomenon has been observed in *Aspergillus oryzae*, where the overexpression of a transcriptional regulator was found to trigger a naturally silent polyketide biosynthetic gene cluster, leading to the production of orthosporin [75]. To date, the biofunctional properties of orthosporin have not been characterized in any organisms. Orthosporin is inferred to be a potential virulence factor, based on the known bioactivities of other isocoumarins. Studies have shown that isocoumarin and its derivatives possess antibacterial, antifungal, and insecticidal activities [39]. The inhibitory effects of isocoumarin on cancer cells, human leukocyte elastase, and blood coagulation have been confirmed in clinical trials [40]. Isocoumarin is also highly effective in inhibiting calpains and serine proteases [76]. In invertebrates that rely on the innate rather than the acquired immune systems, serine proteases are known to be involved in pattern recognition receptor (PRR)-mediated defense responses. When a PRR senses a pathogen-associated molecular pattern such as bacterial polysaccharide and fungal beta-1,3-glucan, it activates serine protease activity, which subsequently initiates a series of immune responses against the pathogen [77]. Serine proteases can regulate various defense reactions in invertebrates, including hemolymph coagulation, antimicrobial peptide synthesis, and melanization. For example, in *Anopheles gambiae*, a serine protease is required for the rapid activation of defense responses against *Plasmodium falciparum* [78]. If orthosporin in the *M. anisopliae* transformant MA05-169 does play a similar role as the isocoumarins in other pathogenic fungi, it may work as an antifungal compound and/or a serine protease inhibitor that attenuates the induced innate immune responses of insects.

In conclusion, the dihydroxynaphthalene-melanin metabolically engineered entomopathogen *M. anisopliae* 05-169 was capable of infecting several important pest insects, including the diamondback moth (*Plutella xylostella*), striped flea beetle (*Phyllotreta striolata*), whitefly (*Bemisia argentifolii*), cowpea aphid (*Aphis craccivora*) and oriental fruit fly (*Bactrocera dorsalis*) across Orders Lepidoptera, Coleoptera, Hemiptera and Diptera Orders. Moreover, the transformant showed a significantly shorter median lethal time (LT50) in *Pl. xylostella* and *Ph. Striolata.*, which was correlated with an elevated conidia germination and appressorium formation rate *in vivo* and *in vitro* (Table 3, 4, 6, 7). The enhanced virulence toward the insect hosts may be directly correlated with the production of huge amounts of secondary metabolites melanin [5] and orthosporin which could indirectly be responsible for the notably enhanced conidia germination and appressoria formation rate, and overexpressed transcripts of key hydrolytic enzymes (chitinase, protease and phospholipase), involved in the infection and colonization of insect hosts. Rationally, the synthesized melanin in the transformant will considerably enhance its hydrophobic attraction force and water absorption capacity, which not only consolidate the adhesion of the hydrophobic conidia to hydrophobic insect host cuticle, but also enhance conidia germination and desiccation tolerance. In addition, melanin plays multiple roles in nature. Importantly it can efficiently scavenge the free radicals, such as the reactive oxygen species (ROS) as those generated by H_2_O_2_ and sodium nitroprusside. Thus, the melanized transformants will be more capable of counteracting the oxidative burst exerted by host immune system *in vivo*, thereby enhancing its virulence toward insect hosts. Although the underlying mechanisms responsible for the drastically increasing in the germination and appressorium formation rate , the formation of orthosporin and the overexpression of hydrolytic enzymes encoding transformant genes remain unknown, these superior characteristics combined with an increased environmental stress tolerance and strong root colonization ability will allow the transformant to infect and colonize the insect hosts more effectively [5], [39], [40], [58], [59], [79]–[81], giving it a biocontrol potential for future field applications.
